# Identification and Validation of lncRNA-SNHG17 in Lung Adenocarcinoma: A Novel Prognostic and Diagnostic Indicator

**DOI:** 10.3389/fonc.2022.929655

**Published:** 2022-06-01

**Authors:** Xinyan Li, Yixiao Yuan, Mintu Pal, Xiulin Jiang

**Affiliations:** ^1^ Department of Pharmacy, Putuo Hospital, Shanghai University of Traditional Chinese Medicine, Shanghai, China; ^2^ Department of Thoracic Surgery, The Third Affiliated Hospital of Kunming Medical University, Kunming, China; ^3^ Department of Pharmacology, All India Institute of Medical Sciences (AIIMS) Bathinda, Punjab, India; ^4^ Key Laboratory of Animal Models and Human Disease Mechanisms of Chinese Academy of Sciences & Yunnan Province, Kunming Institute of Zoology, Kunming, China; ^5^ Kunming College of Life Science, University of Chinese Academy of Sciences, Beijing, China

**Keywords:** lung adenocarcinoma, inflammatory response-related gene, DNA methylation, ceRNA, immune cell infiltration, drug sensitivity

## Abstract

**Background:**

Lung cancer has the highest death rate among cancers globally. Accumulating evidence has indicated that cancer-related inflammation plays an important role in the initiation and progression of lung cancer. However, the prognosis, immunological role, and associated regulation axis of inflammatory response-related gene (IRRGs) in non-small-cell lung cancer (NSCLC) remains unclear.

**Methods:**

In this study, we perform comprehensive bioinformatics analysis and constructed a prognostic inflammatory response-related gene (IRRGs) and related competing endogenous RNA (ceRNA) network. We also utilized the Pearson’s correlation analysis to determine the correlation between IRRGs expression and tumor mutational burden (TMB), microsatellite instability (MSI), tumor-immune infiltration, and the drug sensitivity in NSCLC. Growth curve and Transwell assay used to verify the function of SNHG17 on NSCLC progression.

**Results:**

First, we found that IRRGs were significantly upregulated in lung cancer, and its high expression was correlated with poor prognosis; high expression of IRRGs was significantly correlated with the tumor stage and poor prognosis in lung cancer patients. Moreover, Kyoto Encyclopedia of Genes and Genomes (KEGG) enrichment indicated that these IRRGs are mainly involved in the inflammatory and immune response-related signaling pathway in the progression of NSCLC. We utilized 10 prognostic-related genes to construct a prognostic IRRGs model that could predict the overall survival of lung adenocarcinoma (LUAD) patients possessing high specificity and accuracy. Our evidence demonstrated that IRRGs expression was significantly correlated with the TMB, MSI, immune-cell infiltration, and diverse cancer-related drug sensitivity. Finally, we identified the upstream regulatory axis of IRRGs in NSCLC, namely, lncRNA MIR503HG/SNHG17/miR-330-3p/regulatory axis. Finally, knockdown of SNHG17 expression inhibited lung adenocarcinoma (LUAD) cell proliferation and migration. Our findings confirmed that SNHG17 is a novel oncogenic lncRNA and may be a biomarker for the prognosis and diagnosis of LUAD.

**Conclusion:**

DNA hypomethylation/lncRNA MIR503HG/SNHG17/microRNA-330-3p/regulatory axis may be a valuable biomarker for prognosis and is significantly correlated with immune cell infiltration in lung cancer.

## Introduction

Cancer seriously affects the survival and life of patients, especially lung cancer (LC), which is the main contributor to global cancer mortality, causing more than 700,000 deaths every year (1). Recent research shows that cancer is closely related to autoimmunity, and immunotherapy as a new treatment method has received extensive attention in the field of cancer therapy (2). Immune infiltration in the tumor microenvironment (TME) is the basis of immunotherapy and plays a key role in tumorigenesis and development and also affects the clinical prognosis of patients (3). Immune-checkpoint inhibitors targeting PD-1 or PD-L1 have already substantially improved the outcomes of patients with many types of cancer, but only 20%–40% of patients benefit from these therapies (4). Therefore, it will be helpful to improve the effect of immunotherapy, find the indicators of immune infiltration, and explore its possible mechanism.

As one of the significant characteristics of cancer, cancer-related inflammation mainly includes local inflammation and systemic inflammation (5). The local inflammatory response means the inflammatory microenvironment, which can facilitate cancer progression *via* promoting cancer cell angiogenesis and metastasis and altering the sensitivity of tumor cells to chemotherapeutics drugs (6). The systemic inflammatory response, including alteration in neutrophils and lymphocytes numbers and albumin levels, is significantly associated with the response to diverse cancer treatment (7). Emerging evidence has demonstrated that inflammation plays a crucial role in the occurrence and progression of lung cancer. For instance, it has been shown that tobacco smoke could induce the production of chemokine CCL20 and promote lung cancer progression (8). Tumor-derived CXCL1 was reported to boost NSCLC cell proliferation by recruitment of tumor-associated neutrophils (9). LncRNA HULC *via* upregulation of the expression of sphingosine kinase 1 (SPHK1) leads to facilitation of non-small cell lung cancer cell proliferation and inhibits apoptosis (10). It has been demonstrated that CX3CL1, by activating the Src/focal adhesion kinase signaling pathway, results in facilitating lung cancer cell migration and invasion (11). However, the comprehensive analysis of the prognostic value of inflammatory-response-related genes (IRRGs) and its upstream regulatory axis in LUAD has not yet been elucidated.

In the present research, we employ diverse public databases to explore the expression, gene mutation, prognostic significance, immunological role, and the upstream regulatory axis of inflammation-related gene signature (IRRGs) in LUAD. Our results may provide first evidence for prognostic biomarkers and therapeutic targets for LUAD.

## Materials and Methods

### TCGA Datasets

We acquired the gene profiles and clinical survival data of the LUAD samples from The Cancer Genome Atlas (TCGA) database (https://portal.gdc.cancer.gov/) (12). We utilized these data analysis of the correlation between IRRGs expression and relevant clinical information, including pathological stage.

### Analysis of the Expression and Prognosis of IRRGs in Lung Cancer

We utilized the TIMER (https://cistrome.shinyapps.io/timer/) (13), GSCA tools, and GEPIA databases (http://gepia.cancer-pku.cn/) (14) to analyze the expression and gene mutation, prognosis, and tumor stage of IRRGs in lung cancer; the expression of miRNA-330-3p and lncRNAs were analysis by starBase (15). Kaplan–Meier plotter (http://kmplot.com/analysis/) (16) and GEPIA databases (http://gepia.cancer-pku.cn/) were utilized to examine the prognosis of miRNA-330-3p in lung cancer.

### Identification of Differentially Expressed IRRGs

**Figure 1:**
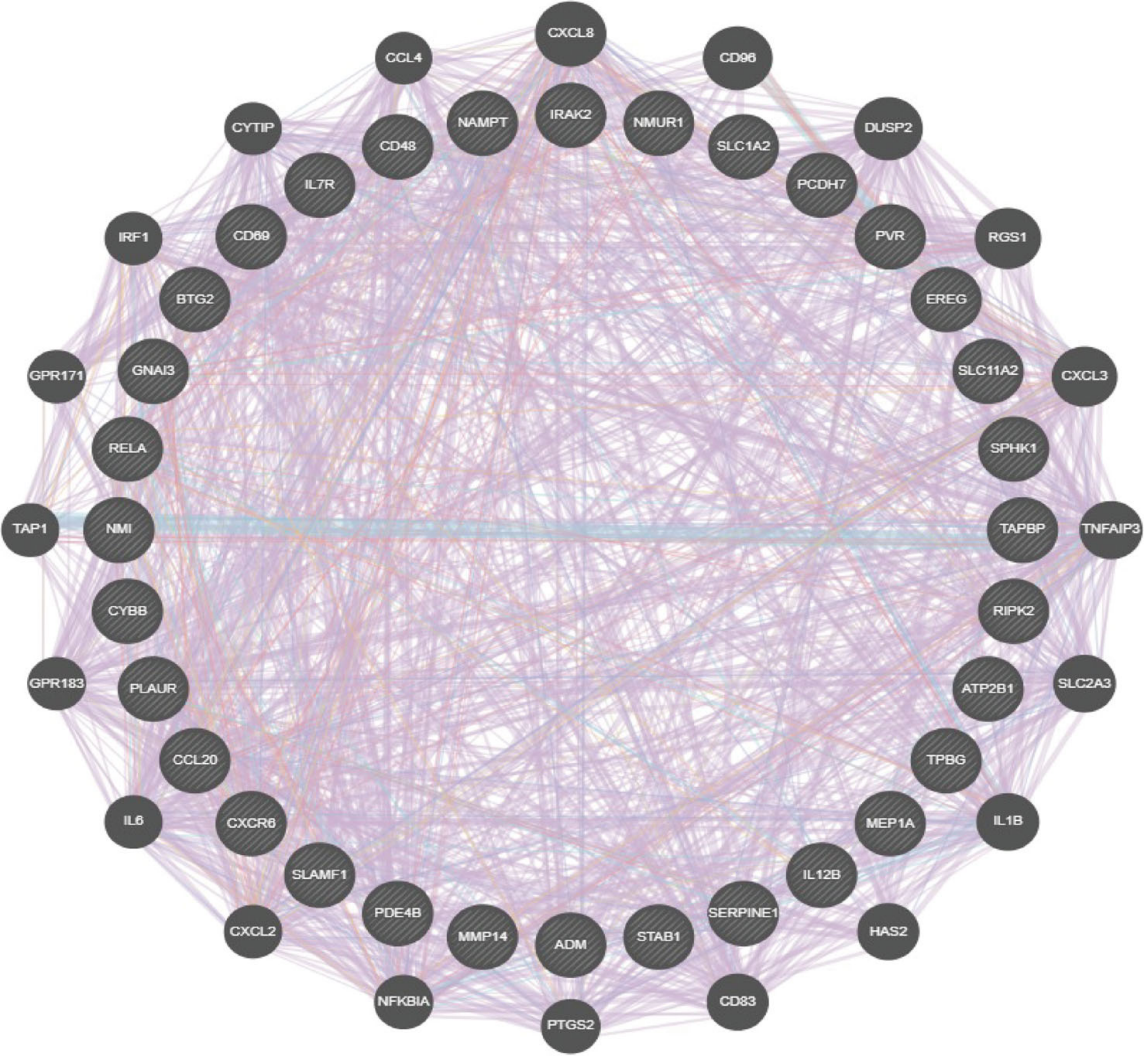
Analysis of the gene and gene interaction network of IRRGs. The gene–gene interaction network of IRRGs was constructed using GeneMania.

A total of 33 IRRGs were obtained from prior reviews (10, 17), which are shown in **Figure 1**. The difference in IRRGs expression in TCGA-LUAD and normal tissues was identified using the “limma” and “reshape2” packages. We then constructed a gene–gene interaction network for 33 IRRGs using the GeneMANIA (http://www.genemania.org) (18).

### Prediction of lncRNA and ceRNA Network Construction

We utilized the starBase database (http://starbase.sysu.edu.cn/), miRDB (http://mirdb.org) (19), miR walk (http://mirwalk.umm.uni-heidelberg.de) (20), and PITA (http://genie.weizmann) (21) to forecast the potential miRNAs of IRRGs (15). We also used the lncBase (www.microrna.gr/LncBase) (22), lncExpdb (https://bigd.big.ac.cn/lncexpdb) (23), and LncRNASNP (http://bioinfo.life.hust.edu.cn/lncRNASNP2) (24) to predicted the potential lncRNAs that binding with miRNA-330-3p, the starBase was employed to examined the expression, prognosis, and correlation between the miRNA-330-3p and lncRNAs; we also used starBase to predict the binding with between miRNA, mRNA, and lncRNA.

### Analysis the Molecular Characteristics of lncRNAs

We employed the lncLocator (www.csbio.sjtu.edu.cn/bioinf/lncLocator.) and CPC2 (http://cpc2.cbi.pku.edu.cn) to examine the subcellular localization and the protein coding ability of lncRNAs (25, 26).

### Functional Enrichment Analysis

Gene Ontology (GO) and Kyoto Encyclopedia of Genes and Genomes (KEGG) pathways were conducted to examine the biological and molecular functions of IRRGs across different cancer types using a total of 300 genes that were positively correlated with IRRGs. All three analyses were performed using the R Package Cluster Profiler.

### Drug Sensitivity Analysis

The relationships between IRRGs expression and sensitivity to drugs were assessed using the Genomics of Drug Sensitivity in Cancer (GDSC) and the Cancer Therapeutics Response Portal (CTRP) databases (27, 28).

### Cancer cells and Cell Culture Conditions

The human bronchial epithelial (BEAS2B) cell line and LUAD cell lines were purchased from the cell bank of Kunming Institute of Zoology and cultured in bronchial epithelial cell growth media (BEGM) (Lonza, Shanghai, CC-3170). HEK-293T was obtained from the American Type Culture Collection (ATCC). Lung cancer cell lines, including H1650, HCC827, and H1975 were purchased from Cobioer (Shanghai, China) with STR document; H1650, HCC827, and H1975 cells were all cultured in Roswell Park Memorial Institute (RPMI) 1640 medium (Corning, Shanghai) supplemented with 10% fetal bovine serum (Cat. No. 10099141C, Gibco, New York, USA) and 1% penicillin/streptomycin.

### SiRNA and Cell Transfection

The siRNA targeting SNHG17 was synthesized (Shanghai Generay Biotech, Shanghai/China). For the transfection of siRNAs and plasmids, cells were transfected using the Lipofectamine 3000 kit according to the manufacturer’s instructions. The sequences for siRNA are as follows: SNHG17 siRNA, GGAGTGTCACATGACTGCCGC.

### Quantitative Real-Time PCR

The quantitative real-time PCR (qRT-PCR) assay was performed as documented (29). The primer sequences are list follows: SNHG17-F, GATTGTCAGCTGACCTCTGTC; SNHG17-R, GTGGTAGCCTCACTCTCCATTCTCTGCCCCT; β-actin-F, CTTCGCGGGCGACGAT; and β-actin-R, CCATAGGAATCCTTCTGACC. The expression quantification was obtained with the 2^−ΔΔCt^ method. Cell proliferation and migration assay was performed as previously documented (30).

### Cell Migration Assay

For the Transwell migration assay, 2.5×10^4^ cells/well in 100 μl serum-free medium were plated in a 24-well plate chamber insert, and the lower chamber was filled with 10% fetal bovine serum (FBS). After incubation for 24 h, cells were fixed with 4% paraformaldehyde, washed, and then stained with 0.5% crystal violet for further imaging.

### CCK8 Assay

We seeded cells in 96-well plates at 2.5 × 10^3^ per well in 100 μl of complete medium and 10 μl of CCK-8 reagent (RiboBio, Guangzhou, China) for 1 h each day after 3 days of culture. We then used a microplate to measure the absorbance of each well at 450 nm. Each sample was evaluated in triplicate.

### Statistical Analysis

All statistical analyses were performed using R software, and receiver operator characteristic (ROC) curves were used to detect IRRGs cutoff values using pROC packages. For the data regarding the function of IRRGs, GraphPad Prism 7.0 was used for statistical analyses.

## Results

### The Expression Pattern and Prognostic Value of IRRG in NSCLC

To comprehensively analyze the prognostic value of IRRGs in lung cancer, we first performed single-factor prognosis analysis. The results demonstrated that 33 genes (ADM, ATP2B1, CCL20, EREG, GNAI3, IRAK2, MMP14, NAMPT, NMI, PCDH7, PLAUR, PVR, RELA, RIPK2, SERPINE1, SPHK1, TAPBP, TPBG, BTG2, CD48, CD69, CXCR6, CYBB, IL7R, IL12B, MEP1A, NMUR1, PDE4B, SLAMF1, SLC1A2, SLC11A2, and STAB1) were significantly related to the prognosis of lung cancer patients. The gene interaction network established by utilized GeneMANIA database (**Figure 1**). We also analyzed the correlation between diverse IRRGs expression in LUAD; results suggested that IRRGs expression was significantly positively associated with IRRGs expression gene in NSCLC (**Supplementary Figure S1**).

**Figure 2:**
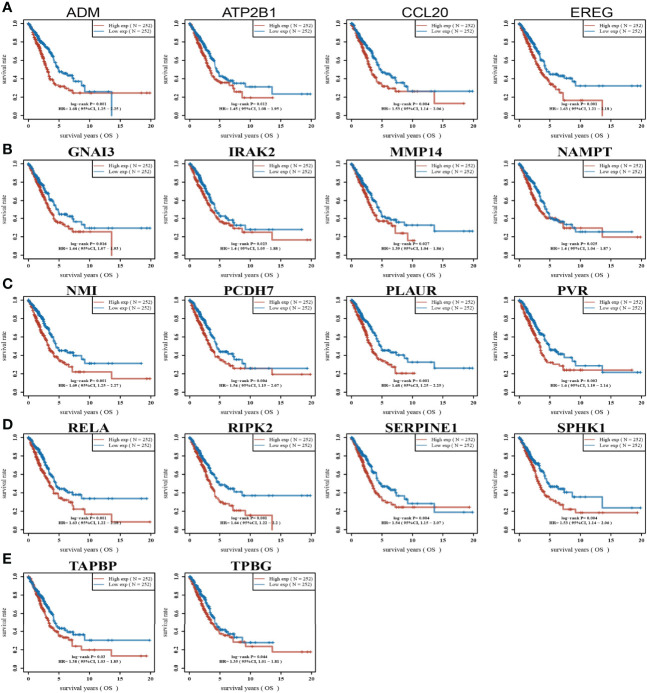
Analysis of the overall survival of IRRGs in LUAD. **(A–E)** The overall survival of IRRGs in LUAD by using the TCGA-LUAD dataset.

**Figure 3:**
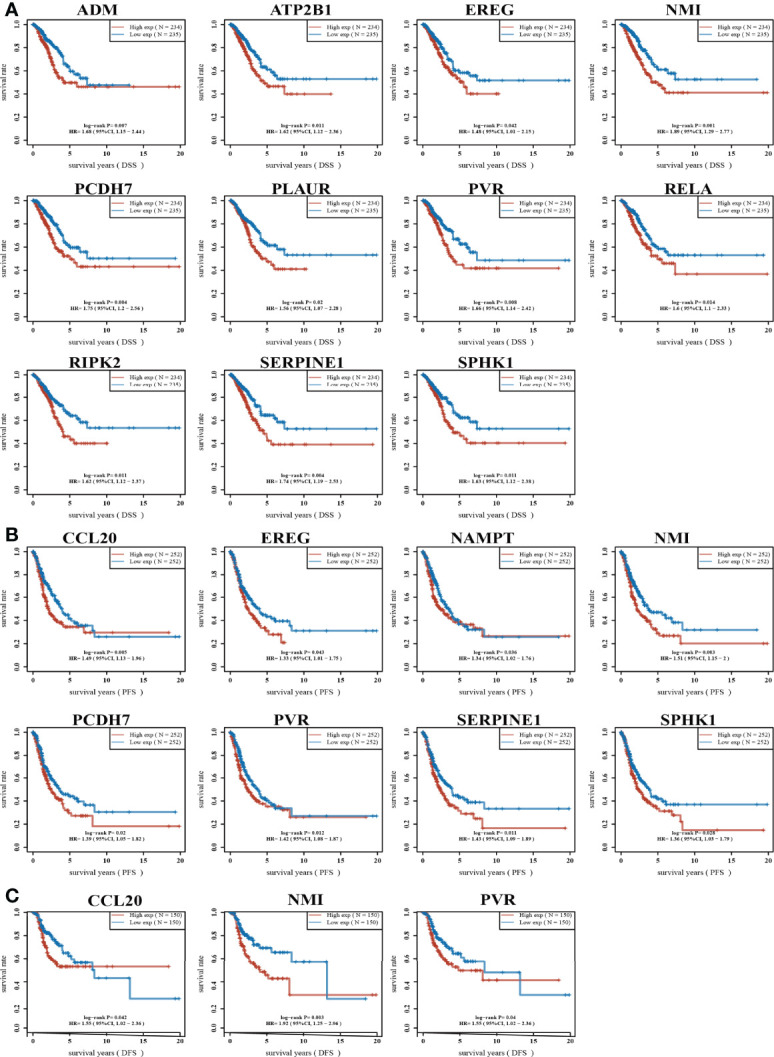
Analysis of the DSS and PFS of IRRGs in LUAD. **(A)** The disease-specific survival of IRRGs in LUAD by using the TCGA-LUAD dataset. **(B)** The progression-free survival of IRRGs in LUAD by using the TCGA-LUAD dataset. **(C)** The disease-free survival of IRRGs in LUAD by using the TCGA-LUAD dataset.

**Figure 4:**
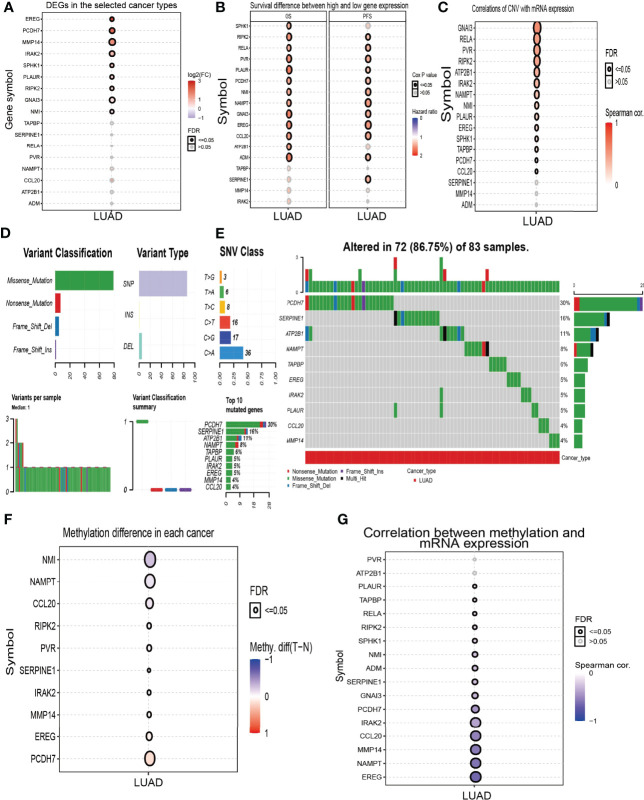
Analysis of the expression and gene mutation of IRRGs in LUAD. **(A)** The expression of IRRGs in LUAD by GSCA tools. **(B)** Analysis the prognosis of IRRGs in LUAD by GSCA tools. **(C)** The correlation between the CNV and IRRGs mRNA expression in LUAD by the GSCA tools. **(D, E)** The mutation frequency and classification of IRRGs in LUAD by the GSCA tools. **(F)** The methylation level in LUAD by the GSCA tools. **(G)** The correlation between the methylation and IRRGs mRNA expression in LUAD by the GSCA tools.

High expression of genes (ADM, ATP2B1, CCL20, EREG, GNAI3, IRAK2, MMP14, NAMPT, NMI, PCDH7, PLAUR, PVR, RELA, RIPK2, SERPINE1, SPHK1, TAPBP, and TPBG) had shorter survival times in lung cancer; this result was verified by Gene Expression Omnibus (GEO) datasets (**Figures 2A–E**; **Supplementary Table S1**). On the contrary, low expression of genes (BTG2, CD48, CD69, CXCR6, CYBB, IL7R, IL12B, MEP1A, NMUR1, PDE4B, SLAMF1, SLC1A2, SLC11A2, and STAB1) had shorter survival times in lung cancer (**Supplementary Figure S2**). Additionally, we found that these genes (ADM, ATP2B1, EREG, NMI, PCDH7, PLAUR, PVR, RELA, RIPK2, SERPINE1, SPHK1) correlated with poor disease-specific survival (DSS) in lung cancer (**Figure 3A**), high expression of CCL20, EREG, NAMPT, NMI, PCDH7, PVR, SERPINE1, and SPHK1 correlated with poor progression-free survival in patients with lung cancer and high expression of CCL20, NMI, and PVR associated with poor disease-free survival (DFS) in patients with lung cancer (**Figures 3B, C**). High expression predicted poor prognosis and shows that these genes may play oncogenic roles in the progression of lung cancer. Therefore, we chose these 18 genes to further analyze the function. Next, we determined the expression of IRRGs in NSCLC by employing the GSCA database that is based on TCGA LUAD datasets. The results verify that 10 genes were upregulated in lung cancer; other genes had no significant difference in lung cancer (**Figures 4A, B**).

### Gene Mutation Analysis of IRRGs in NSCLC

The copy number variation (CNV) of the gene usually leads to its overexpression in diverse cancer. Based on the conclusion, we first analyzed the CNV of IRRGs. The results demonstrated that the genes’ (GNAI3, RELA, PVR, RIPK2, ATP2B1, IRAK2, NAMPT, NMI, PLAUR, and EREG) CNV was significantly associated with the expression of mRNA in LUAD (**Figure 4C**). We then summarize the incidence of copy number variations and somatic mutations of 18 IRRGs in LUAD. Seventy-two of 83 (86.75%) LUAD samples demonstrated genetic mutations (**Figures 4D, E**). A missense mutation was the most common variant classification (**Figures 4D, E**). SNPs were the most common variant type, and C > A and C>G ranked as the top SNV class (**Figures 4D, E**). Results confirmed *PCDH7* as the gene with the highest mutation frequency (**Figures 4D, E**). Next, we analyzed the DNA methylation information for 18 IRRGs in LUAD. Results demonstrated that the methylation levels of NMI, NAMPT, and CCL20 were decreased in LUAD than in the normal group (**Figure 4F**). On the contrary, the methylation levels of EREG and PCDH7 were increased in LUAD (**Figure 4F**); the methylation of IRRGs was significantly negatively correlated with the expression of IRRGs in the progression of LUAD (**Figure 4G**). Collectively, these results suggested that CNV and DNA methylation significantly affected the expression of IRRGs in lung cancer.

### GO and KEGG Enrichment for IRRGs in NSCLC

**Figure 5:**
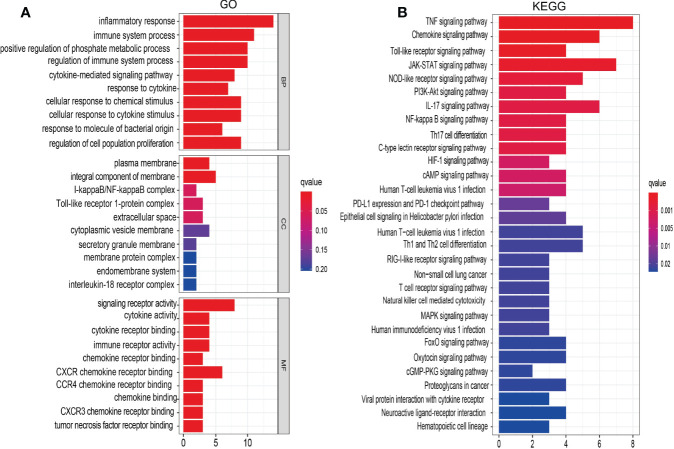
Analysis of the functions of IRRGs in LUAD. **(A)** The GO enrichment terms are involved by IRRGs in LUAD. **(B)** The KEGG pathway is involved by IRRGs in LUAD.

To explore the potential function of IRRGs in the NSCLC, we conducted the GO and KEGG enrichment analysis. Results indicated that these 18 IRRGs mainly participated in the biological process such as the inflammatory response and regulation of cell population proliferation in GO term (**Figure 5A**). In molecular functions of GO term, these 18 IRRGs were mainly involved in the signaling receptor activity, cytokine receptor binding, cytokine activity, CXCR3 chemokine receptor binding, and tumor necrosis factor receptor binding in GO term (**Figure 5A**). We observed that these 18 IRRGs were mainly involved in cytoplasmic vesicle membrane, secretory granule membrane, membrane protein complex, endomembrane system, and interleukin-18 receptor complex in cell component of GO terms (**Figure 5A**). Furthermore, KEGG pathway enrichment results indicated that 18 IRRGs were mainly involved in the PI3K-Akt signaling pathway, IL-17 signaling pathway, nuclear factor (NF)-kappa B signaling pathway, Th17 cell differentiation, C-type lectin receptor signaling pathway, hypoxia-inducible factor 1 (HIF-1) signaling pathway, cAMP signaling pathway, epithelial cell signaling in *Helicobacter pylori* infection, Th1 and Th2 cell differentiation, mitogen-activated protein kinase (MAPK) signaling pathway, human immunodeficiency virus 1 infection, FoxO signaling pathway, oxytocin signaling pathway, cGMP-PKG signaling pathway, proteoglycans in cancer, neuroactive ligand–receptor interaction, and hematopoietic cell lineage (**Figure 5B**). Collectively, these data imply that IRRGs affect the inflammation and immune responses and participated in the progression of NSCLC.

### Correlation Between IRRGs Expression and Pathological Stage in NSCLC

**Figure 6:**
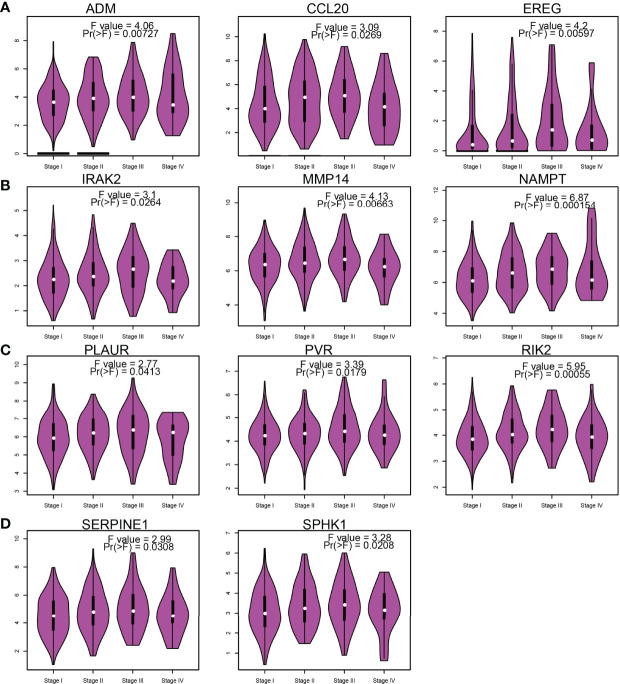
Analysis of the tumor stage of IRRGs in LUAD. **(A–D)** The tumor stage of IRRGs in LUAD by using GEPIA database.

Considering that the IRRGs were upregulated in NSCLC, we further examined the correlation between IRRGs expression and pathological stage of NSCLC. Results confirmed that ADM, CCL20, EREG, IRAK2, MMP14, NAMPT, OLAUR, PVR, RIK2, SERPINE1, and SPHK1 expressions were significantly related to the pathological stage in NSCLC (**Figures 6A–D**).

### Construction of Inflammatory Response-Related Gene Prognostic Model

**Figure 7:**
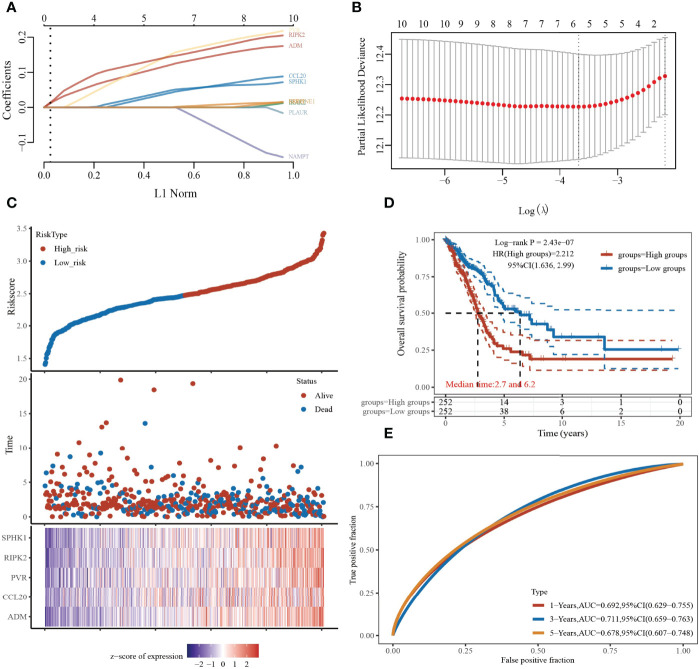
Construction of a prognostic IRRGs model in LUAD. **(A)** LASSO coefficient profiles of the 10 IRRGs. **(B)** Plots of the 10-fold cross-validation error rates. **(C)** Distribution of risk score, survival status, and the expression of five prognostic IRRGs in LUAD. **(D, E)** Overall survival curves for LUAD patients in the high-/low-risk group and the ROC curve of measuring the predictive value.

To construct a prognostic gene model, we employed the univariate Cox regression analysis to screen those IRRGs with a prognostic value. As is shown in **Figures 7A, B**, LASSO Cox regression analysis was performed to construct a prognostic gene model based on these 10 prognosis IRRGs (**Figures 7A, B**). The risk score=(0.1198)*ADM+ (0.0404)*CCL20+ (0.1478)*PVR+ (0.144)*RIPK2+ (0.047)*SPHK1. Based on the risk score, LUAD patients were divided into two groups. The higher risk group had shorter survival times in patients with LUAD (**Figures 7C, D**), with areas under the curve (AUCs) of 0.692, 0.711, and 0.678 in the 1-, 3-, and 5-year ROC curves, respectively (**Figure 7E**).

### Building a predictive nomogram

**Figure 8:**
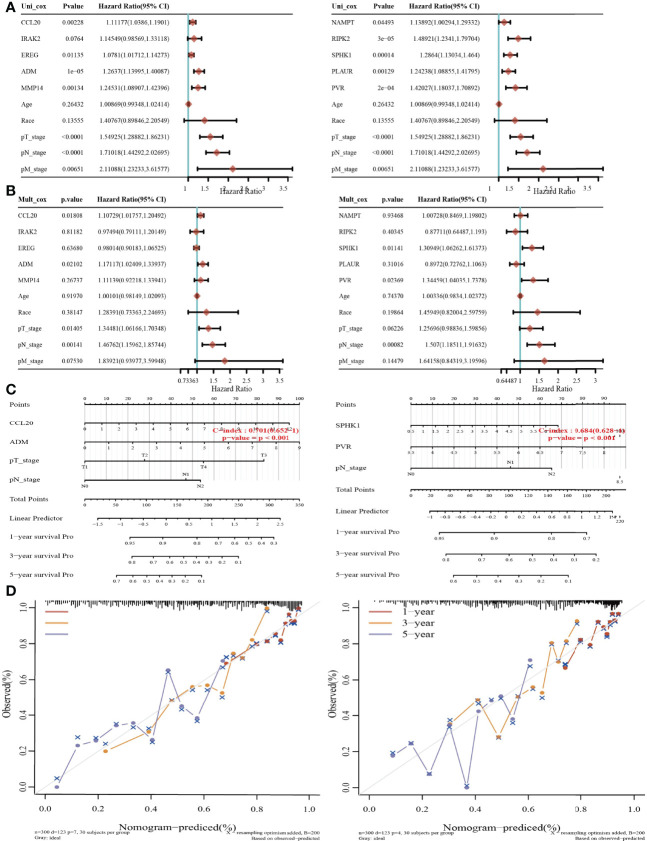
Construction of a predictive nomogram in LUAD. **(A, B)** Hazard ratio and p‐value of the constituents involved in univariate and multivariate Cox regression considering clinical parameters and five prognostic IRRGs in LUAD. **(C, D)** Nomogram to predict the 1-, 3-, and 5-year overall survival rate of LUAD patients. Calibration curve for the overall survival nomogram model in the discovery group.

Considering that these six prognostic IRRGs were correlated with the tumor stage in LUAD, we next constructed a predictive nomogram to predict the survival state. Univariate and multivariate analyses revealed that CCL20, ADM, or SPHK1 expression and T, N, and M stages were independent factors affecting the prognosis of LUAD patients (**Figures 8A, B**). The predictive nomogram confirmed that the 1-, 3-, and 5-year overall survival rates could be predicted relatively well compared with an ideal model in the entire cohort (**Figures 8C, D**). ROC curve analysis results confirmed that ADM, IRAK2, and MMP14 may be biomarkers in NSCLC with high sensitivity and specificity (**Supplementary Figure S3**).

### Associations Between IRRGs Expression and TMB, MSI, and Drug Sensitivity

**Figure 9:**
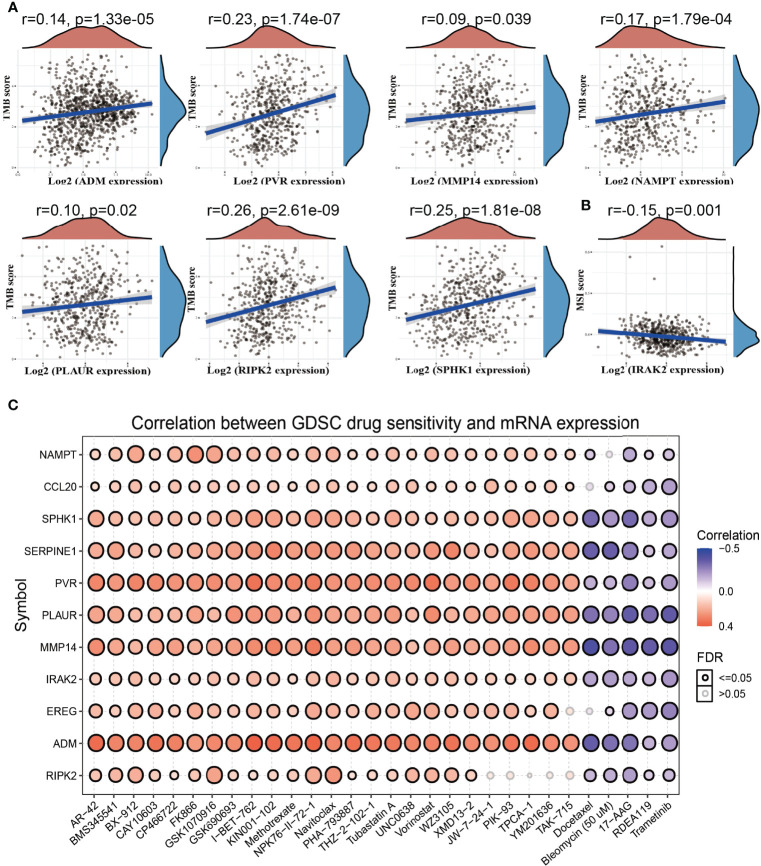
Analysis of the correlation between the IRRGs expression and TMB, MSI, and drug sensitivity. **(A)** The correlation between the IRRGs expression and TMB in LUAD. **(B)** The correlation between the IRAK2 expression and MSI in LUAD. **(C)** The correlation between the IRRGs expression and drug sensitivity in the GDSC database.

Emerging evidence has demonstrated that tumor mutational burden (TMB) and microsatellite instability (MSI) could be a potential biomarker for predicting the efficacy of immunotherapy for lung cancer (29). The above findings indicated that the IRRGs were related to tumor immune infiltration. To determine the relationships between IRRG expression and TMB, MSI, and drug sensitivity in NSCLC, we conducted the related correlation analysis. The analysis revealed that among these IRRGs, ADM, PVR, MMP14, PLAUR, and RIPK2 were positively correlated with the TMB (**Figure 9A**), while IRAK2 was negatively correlated with the MSI (**Figure 9B**). To explore the potential therapy target, it is extremely important to examine the correlation between these IRRGs expressions and diverse drugs in lung cancer. In the present study, we employed the GSCA tools to analyze the relationship between the IRRGs expression and drug sensitivity. The results demonstrated that ADM and CCL20 expression was positively correlated with the drug sensitivity of I-BET-762, NPK76-II-72-1, TPCA-1, KIN001-102, AR-42, and PHA-793887 (r>0.34, p<0.001) and negatively associated with the drug sensitivity of Docetaxel, Bleomycin (50 μM), 17-AAG, Dasatinib, and TGX221; CCL20, IRAK2,NAMPT, PVR, SERPINE1, and SPHK1 were correlated with the drug sensitivity of diverse drug (**Figure 9C**).

### IRRGs Were Associated With Tumor Immune Infiltration in LUAD

We used TIMER database analysis and found that the somatic copy number alterations (SCNA) of IRRGs were related to diverse immune cell infiltration levels in LUAD (**Supplementary Figure S4**). Next, we analyzed the expression of IRRGs (ADM, CCL20, EREG, IRAK2, MMP14, NAMPT, PLAUR, PVR, RIPK2, SERPINE1, and SPHK1) in an immune subtype of LUAD. The results demonstrated that ADM was mainly highly expressed in the C4 subtype, CCL20 was mainly highly expressed in C1 subtype, EREG was mainly high expressed in C2 subtype, IRAK2 was mainly highly expressed in C4 subtype, MMP14 was mainly highly expressed in C6 subtype, NAMPT was mainly highly expressed in C1 subtype, PLAUR was mainly highly expressed in C1 subtype, PCR was mainly highly expressed in C1 subtype, RIK2 was mainly highly expressed in C2 subtype, SERPINE1 was mainly highly expressed in C6 subtype, and SPHK1 was mainly highly expressed in C2 subtype (**Supplementary Figure S5**).

**Figure 10:**
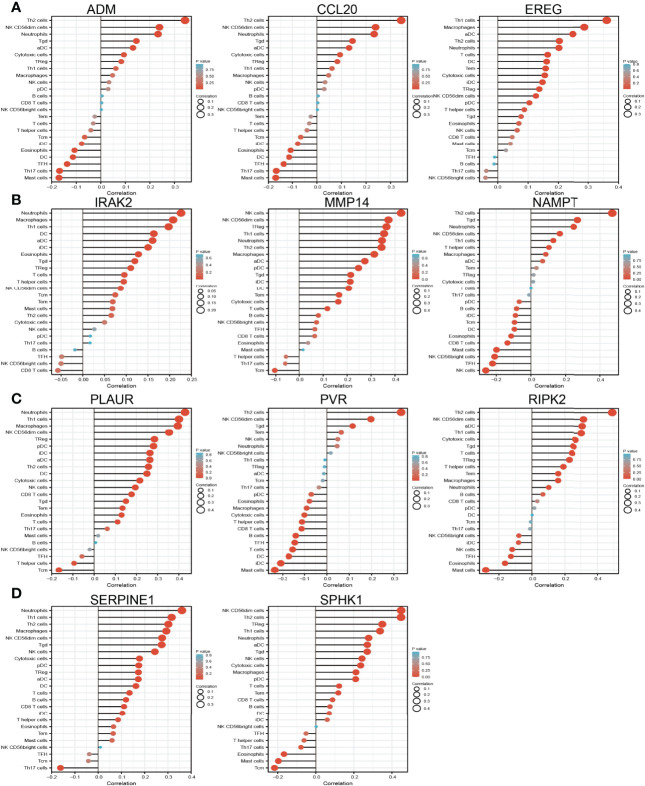
Analysis of the association between IRRGs expression and immune infiltration level in LUAD. **(A–D)** The association between IRRGs expression and immune infiltration level in LUAD.

Considering that inflammation plays crucial roles in the immune response and development and progression of lung cancer (31), we explored the correlation between the expression of IRRGs (ADM, CCL20, EREG, IRAK2, MMP14, NAMPT, PLAUR, PVR, RIPK2, SERPINE1, and SPHK1) and immune infiltration in LUAD by employing the TIMER database. The analysis data demonstrated that IRRGs were positively or negatively correlated with the immune infiltration of Th2 cells, NK, CD56dim cells, neutrophils, Tgda, DC, cytotoxic cells, and TReg, FH, Th17 cells, and mast cells (**Figures 10A–D**).

### Analysis the Upstream Molecular Regulatory Axis of IRRGs

**Figure 11:**
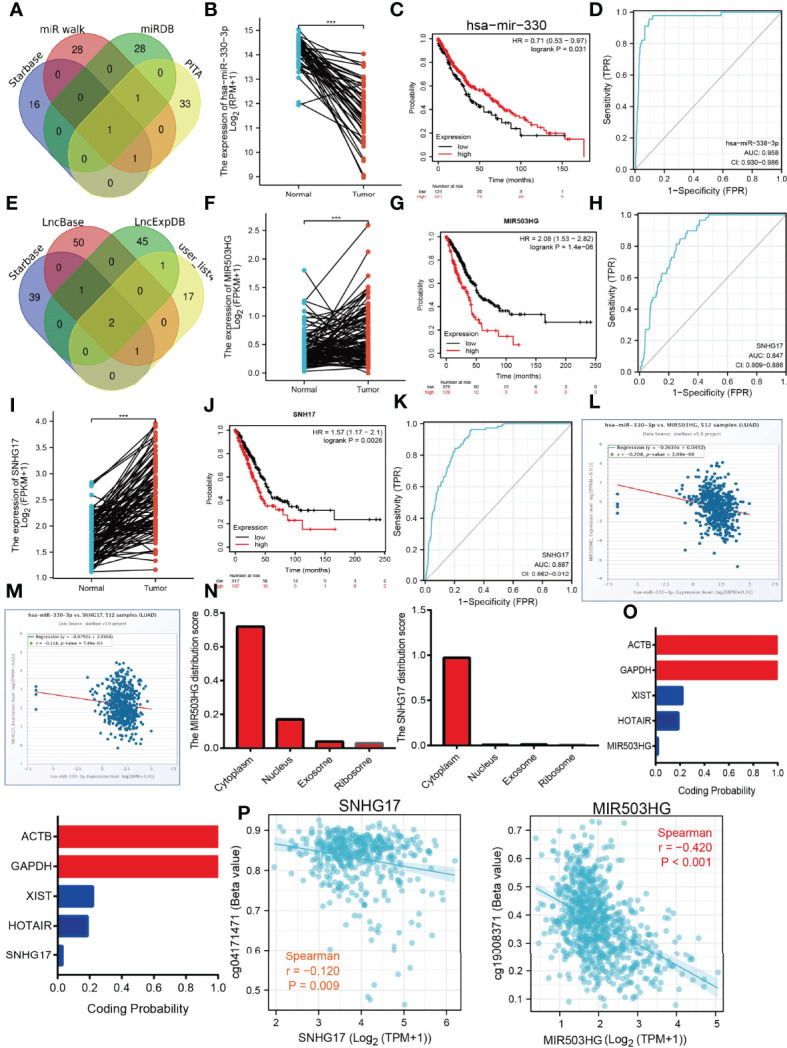
Construction of lncRNA/miRNA/IRRGs interaction network. **(A)** Predict the potential miRNAs of IRRGs in LUAD examined by using starBase. **(B)** The expression of miRNA-330-3p in LUAD by using starBase.**(C)** The prognosis of miRNA-330-3p in LUAD by kmplot database. **(D)** ROC curve analyses and AUC values for miRNA-330-3p in LUAD. **(E)** Predict the potential lncRNAs of miRNA-330-3p in LUAD by using starBase. **(F)** The expression of MIR530HG in LUAD by using starBase. **(G)** The prognosis of MIR530HG in LUAD by kmplot database. **(H)** ROC curve analyses and AUC values for MIR530HG in LUAD. **(I)** The expression of SNHG17 in LUAD by using starBase. **(J)** The prognosis of SNHG17 in LUAD by using kmplot database. **(K)** ROC curve analyses and AUC values for SNHG17 in LUAD. **(L, M)** Pearson’s correlation analysis determined the relationship between miRNA-330-3p and MIR530HG1 and SNHG17 expression in LUAD examined by using starBase. **(N)** The subcellular location of MIR530HG1 and SNHG17 by the lncLocator and Annolnc2 databases. **(O)** The coding ability of MIR530HG1 and SNHG17 by the coding potential calculator databases. **(P)** Correlation between DNA methylation and MIR530HG1, SNHG17 expression in LUAD. ***P < 0.001.

The above results suggested that ADM, CCL20, EREG, IRAK2, MMP14, PVR, RIPK2, and SPHK1 expressions were correlated with the tumor stage in the LUAD, another gene with no significant correlation. This evidence indicated that ADM, CCL20, EREG, IRAK2, MMP14, PVR, RIPK2, and SPHK1 may participate in the cancer progression of LUAD. To further explore the upstream molecular regulatory axis of IRRGs, we employed diverse public databases to construct a network of mRNA–miRNA–lncRNA interactions. These data indicate that miRNA-330-3p is the most potential miRNA that binds with the 3′-untranslated region (3′UTR) of ADM, CCL20, EREG, IRAK2, MMP14, PVR, RIPK2, and SPHK1 (**Figure 11A**). Further analysis found that miRNA-330-3p was decreased in the lung cancer, and low expression of miRNA-330-3p correlated with the unfavorable prognosis of lung cancer patients (**Figures 11B, C**). ROC curve analysis of miRNA-330-3p showed an AUC value of 0.958 in lung cancer patients (**Figure 11D**). Next, we further explored its upstream lncRNA targets to construct the miRNA–lncRNA axis. We employed the starBase, lncBase, lncExpdb, and LncRNASNP for prediction and obtained two lncRNAs, including the MIR503HG and SNHG17 (**Figure 11E**). Further study demonstrated that MIR503HG and SNHG17 were upregulated in NSCLC, and high expression was correlated with poor prognosis in LUAD. ROC curve analysis of MIR503HG and SNHG17 showed an AUC value of 0.847 and 0.887 in lung cancer patients, respectively (**Figures 11F–K**). According to the competing endogenous RNA (ceRNA) theory, the lncRNA should be negative and positively correlated with the expression of miRNA and mRNA, respectively. We further study found that MIR503HG and SNHG17 were negatively associated with the expression of miRNA-330-3p **(** **Figures 11L, M**). Moreover, we also showed that MIR503HG and SNHG17 primarily localized in the cytoplasm examined by using the lnclocator and annolncRNA tools (**Figure 11N**) and did not possess the coding potential (**Figure 11O**). We also uncovered that the methylation levels on the specific methylation sites (cg19003871 and cg04171471) within the MIR503HG and SNHG17 promoter region negatively correlated with its expression in lung cancer (**Figure 11P**). Collectively, these data indicate that the lncRNA MIR503HG/SNHG17/miR-330-3p/IRRGs regulatory axis may play crucial role in the progression of LUAD.

### SNHG17 Inhibits Cancer Cell Migration and Invasion

**Figure 12:**
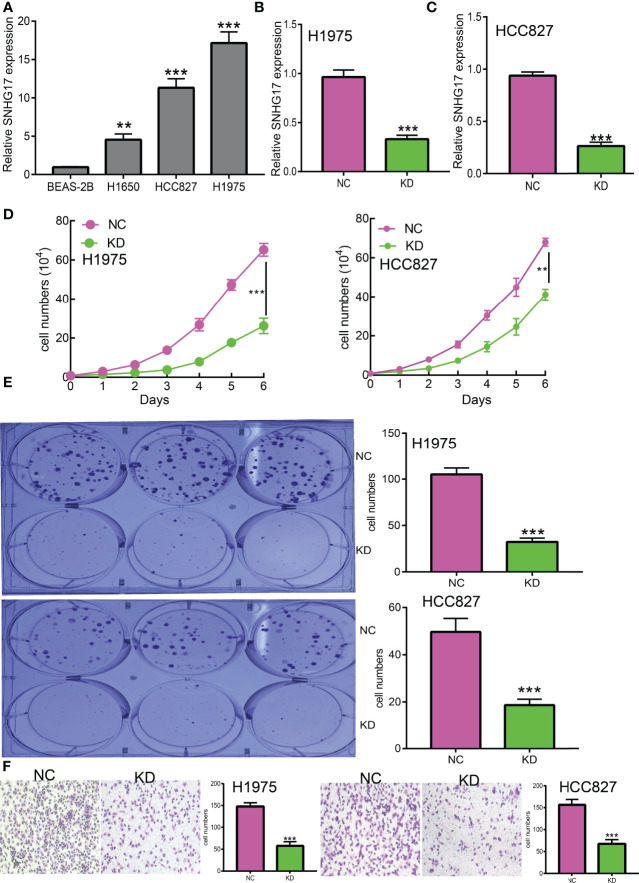
SNHG17 promotes LUAD cell proliferation, migration, and invasion *in vitro.*** (A)** The relative expression level of SNHG17 in lung adenocarcinoma cancerous cell lines, including H1650, HCC827, and H1975 examined by real-time RT-PCR, compared to normal human bronchial epithelial cell line: BEAS-2B. **(B,C)** The establishment of SNHG17 knockdown cell lines in HCC827 and H1975 verified by real-time RT-PCR **(D, E)** knockdown of SNHG17 significantly inhibits cell proliferation in HCC827 and H1975 cells, as measured by growth curve and colony formation assay; scale bar, 50 μM. **(F)** Knockdown of SNHG17 dramatically inhibits HCC827 and H1975 cells migration ability examined by Transwell. **P < 0.01, ***P < 0.001.

Currently, there are still no studies examining whether SNHG17 is correlated with cancer progression. We decided to investigate the functional roles of SNHG17 in LUAD. We found that SNHG17 was increased in the LUAD cell lines (H1650, HCC827, and H1975) compared with that in the normal lung epithelial cell line (BEAS2B) (**Figure 12A**), which is consistent with the online database that we discovered. Given that SNHG17 was upregulated in LUAD, we then inhibited the SNHG17 expression using siRNA, and the knockdown efficiency of SNHG17 was verified by real-time RT-PCR assay **(** **Figures 12B, C**). Then, we evaluated the effects of SNHG17 on LUAD cell proliferation and migration capacities by growth curve, colony formation, and Transwell assays. We showed that downregulation of SNHG17 significantly decreased the proliferation and migratory capabilities of LUAD cells (**Figures 12D–F**). Collectively, these results confirmed that SNHG17 was highly expressed in LUAD cells and significantly affected their migration and invasion.

## Discussion

TME plays an important role in the dynamic regulation of tumor progression, and strategies to therapeutically target the TME have emerged as a promising approach for cancer treatment^23^. Immunotherapy has been approved in clinical or being evaluated in trials and has a wide application prospect (17). Immune cells include T and B lymphocytes, tumor-associated macrophages, dendritic cells (DCs), natural killer cells, neutrophils, and myeloid-derived suppressor cells (MDSCs) (32). A comprehensive analysis of tumor-infiltrating immune cells will help to clarify the mechanism of tumor immune escape and provide opportunities for the development of new therapeutic strategies. It has been well recognized that cancer-related inflammatory plays crucial roles in the initiation and progression of lung cancer. However, the prognosis significance and regulatory mechanism of inflammatory response-related genes in lung cancer remain unclear.

We first determine the expression pattern and prognostic value of IRRGs in LUAD. We found that the expressions of ADM, ATP2B1, CCL20, EREG, GNAI3, IRAK2, MMP14, NAMPT, NMI, PCDH7, PLAUR, PVR, RELA, RIPK2, SERPINE1, SPHK1, TAPBP, and TPBG were increased, while the expressions of BTG2, CD48, CD69, CXCR6, CYBB, IL7R, IL12B, MEP1A, NMUR1, PDE4B, SLAMF1, SLC1A2, SLC11A2, and STAB1 were downregulated in LUAD. Prognosis analysis suggested that high expressions of ADM, ATP2B1, CCL20, EREG, GNAI3, IRAK2, MMP14, NAMPT, NMI, PCDH7, PLAUR, PVR, RELA, RIPK2, SERPINE1, SPHK1, TAPBP, and TPBG were correlated with poor overall survival and tumor stage in LUAD. These data suggested that the expression of *IRRGs* was associated with the prognosis of LUAD. Additionally, we found that the CNV and DNA methylation level of *IRRGs* was significantly positively and negatively correlated with the expression of *IRRGs* in LUAD.

The functional enrichment analysis of IRRGs showed that these 33 IRRGs were mainly involved in the PI3K-Akt signaling pathway, IL-17 signaling pathway, NF-kappa B signaling pathway, Th17 cell differentiation, C-type lectin receptor signaling pathway, and HIF-1 signaling pathway.

LASSO Cox regression analysis was performed to construct a prognostic gene model based on 10 prognostic IRRGs (CCL20, IRAK2, EREG, ADM, MMP14, NAMPT, RIPK2, SPHK1, PLAUR, and PVR), which could predict the overall survival of LUAD patients with medium to high accuracy. In our study, CCL20, IRAK2, EREG, ADM, MMP14, NAMPT, RIPK2, SPHK1, PLAUR, and PVR could be effective gene signatures for predicting the prognosis of LUAD. It has been documented that CCL20 production from lung cancer inflammatory microenvironment, by activation PI3K signaling pathway, led to the boosting of the cell migration and proliferation of NSCLC cell lines (33). Previous studies demonstrated that a genetic variant of IRAK2 may be a promising prognostic biomarker for NSCLC OS (34). Another study demonstrated that SphK1 elevated the expression of STAT3 and facilitated the progression of non−small-cell lung cancer (35). Overexpression of PLAUR confers resistance to gefitinib *via* activating the EGFR/P-AKT signaling pathway in NSCLC (36). A recent study has shown that PVR has the potential value for predicting the prognosis of these patients (37).

Emerging evidence has demonstrated that the tumor microenvironment plays crucial roles in tumor proliferation, angiogenesis, invasion, and metastasis, and chemotherapeutic resistance. In this study, we found that IRRGs expression had significant correlation with the immune infiltration of diverse immune cells. We also found that ADM, PVR, MMP14, PLAUR, and RIPK2 were positively correlated with the TMB. On the contrary, IRAK2 was negatively correlated with the MSI. These results demonstrate that IRRGs play crucial roles in immune response in lung cancer.

**Figure 13:**
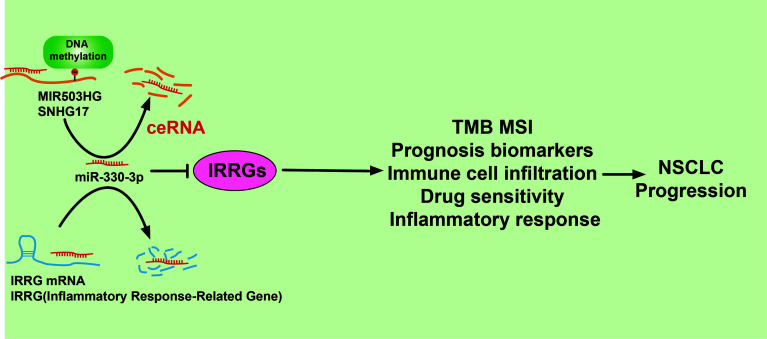
A working model for lncRNA MIR530HG1/SNHG17/miR-330-3p/IRRGs axis in LUAD.

We also use the diverse public databases to construct a new mRNA–miRNA–lncRNA network that modulates the expression of IRRGs *via* ceRNA manner, that is, lncRNA MIR503HG and SNHG17/miR-330-3p/IRRGs axis. Previous studies reported that MIR503HG was highly expressed in NSCLC tissues than in adjacent tissues. Depletion of lncRNA MIR503HG significantly inhibits the cell proliferation and facilitates cell apoptosis of NSCLC cells *via* downregulation of the expression of miR-489-3p and miR-625-5p (38). Similarly, it was confirmed that lncRNA SNHG17 modulates the miR-449a/TGIF2 axis and promotes NSCLC cell proliferation, migration, invasion, and epithelial to mesenchymal transition (39). On the contrary, it has been suggested that microRNA-330-3p inhibits the NSCLC progression by inhibiting the expression of GRIA3 (40). In our study, we showed that high expression of MIR503HG/SNHG17 and low expression of microRNA-330-3p were correlated to poor prognosis of LUAD patients. As a crucial epigenetic mechanism, DNA methylation plays major roles in the regulation gene expression. In our finding, we confirm that hypomethylation for the promoter of MIR503HG/SNHG17 results in its overexpression in lung cancer. This evidence indicated that the DNA hypomethylation/lncRNA MIR503HG/SNHG17/microRNA-330-3p/IRRGs regulatory axis may play a crucial role in the progression of LUAD (**Figure 13**). Currently, there are still no studies examining whether SNHG17 is correlated with cancer progression. We found that downregulation of SNHG17 inhibited tumor cell migration, and cell invasion.

## Conclusion

Together, these findings suggest that DNA hypomethylation/lncRNA MIR503HG/SNHG17/microRNA-330-3p/inflammatory response-related gene (IRRG) signature is a valuable biomarker for prognostic and significantly correlated with immune infiltration in lung cancer.

## Data Availability Statement

The original contributions presented in the study are included in the article/**Supplementary Material**. Further inquiries can be directed to the corresponding authors.

## Author Contributions

XL and YY designed this work and performed related assay and analyzed the data. MP and XJ supervised and wrote the manuscript. All authors contributed to the article and approved the submitted version.

## Funding

This work was supported by the National Nature Science Foundation of China (82160512, 30960398, 81460174, 81360126), Yunnan Applied Basic Research Projects (2017FE467 and 2018FE001), and the Applied Basic Research Project of Yunnan Provincial Science and Technology Department, Kunming Medical University (No.2020001AY070001-117) and the Open Project of The First People's Hospital of Yunnan Province Clinical Medicine Center (2021LCZXXF‐XZ03).

## Conflict of Interest

The authors declare that the research was conducted in the absence of any commercial or financial relationships that could be construed as a potential conflict of interest.

## Publisher’s Note

All claims expressed in this article are solely those of the authors and do not necessarily represent those of their affiliated organizations, or those of the publisher, the editors and the reviewers. Any product that may be evaluated in this article, or claim that may be made by its manufacturer, is not guaranteed or endorsed by the publisher.
