# Corrigendum: identification and validation of lncRNA-SNHG17 in lung adenocarcinoma: a novel prognostic and diagnostic indicator

**DOI:** 10.3389/fonc.2022.972329

**Published:** 2022-07-26

**Authors:** Xinyan Li, Yixiao Yuan, Mintu Pal, Xiulin Jiang

**Affiliations:** ^1^ Department of Pharmacy, Putuo Hospital, Shanghai University of Traditional Chinese Medicine, Shanghai, China; ^2^ Department of Thoracic Surgery, The Third Affiliated Hospital of Kunming Medical University, Kunming, China; ^3^ Department of Pharmacology, All India Institute of Medical Sciences (AIIMS) Bathinda, Punjab, India; ^4^ Key Laboratory of Animal Models and Human Disease Mechanisms of Chinese Academy of Sciences & Yunnan Province, Kunming Institute of Zoology, Kunming, China; ^5^ Kunming College of Life Science, University of Chinese Academy of Sciences, Beijing, China

**Keywords:** lung adenocarcinoma, inflammatory response-related gene, DNA methylation, ceRNA, immune cell infiltration, drug sensitivity

In the original article, author Mintu Pal was mistakenly affiliated with - “Biotechnology Division, North East Institute of Science and Technology, Jorhat, India” as published. The correct affiliation should be – “Department of Pharmacology, All India Institute of Medical Sciences (AIIMS) Bathinda, Punjab, India” as above.

The authors apologize for this error and state that this does not change the scientific conclusions of the article in any way. The original article has been updated.

## Publisher’s note

All claims expressed in this article are solely those of the authors and do not necessarily represent those of their affiliated organizations, or those of the publisher, the editors and the reviewers. Any product that may be evaluated in this article, or claim that may be made by its manufacturer, is not guaranteed or endorsed by the publisher.

