# Supplementary material for: Identification and Validation of lncRNA-SNHG17 in Lung Adenocarcinoma: A Novel Prognostic and Diagnostic Indicator
Source: Front Oncol. 2022 Jun 1;12:929655. doi: 10.3389/fonc.2022.929655 (PMC9198440; doi:10.3389/fonc.2022.929655)
Supplement: Supplementary file 1 [file DataSheet_1.docx]

Supplementary Material


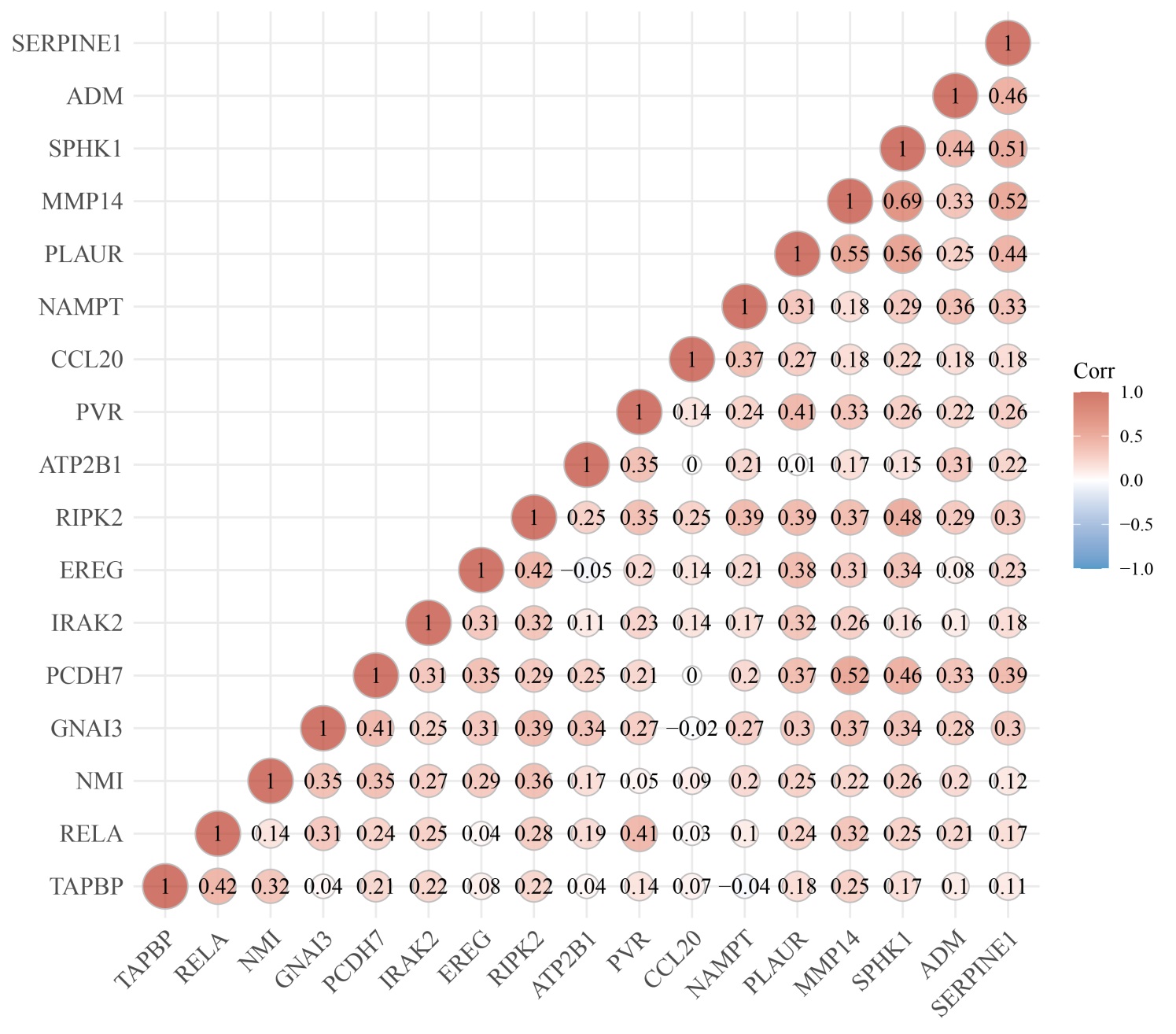


**Supplementary FIGURE 1 ⎜ Correlation between the diverse IRRGs expression in LUAD.**


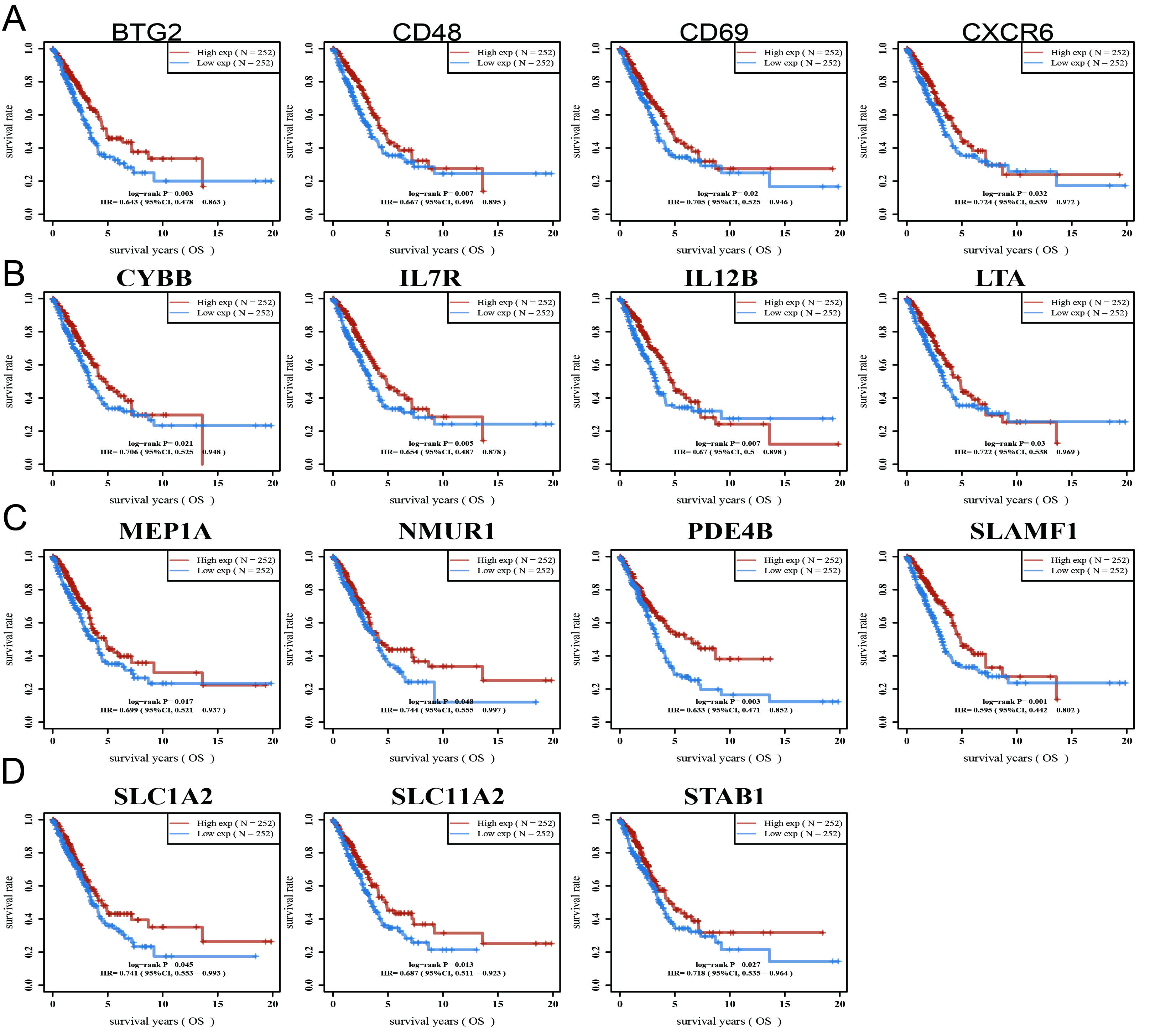


**Supplementary FIGURE 2 ⎜ Analysis of the prognosis of IRRGs in LUAD.**

**
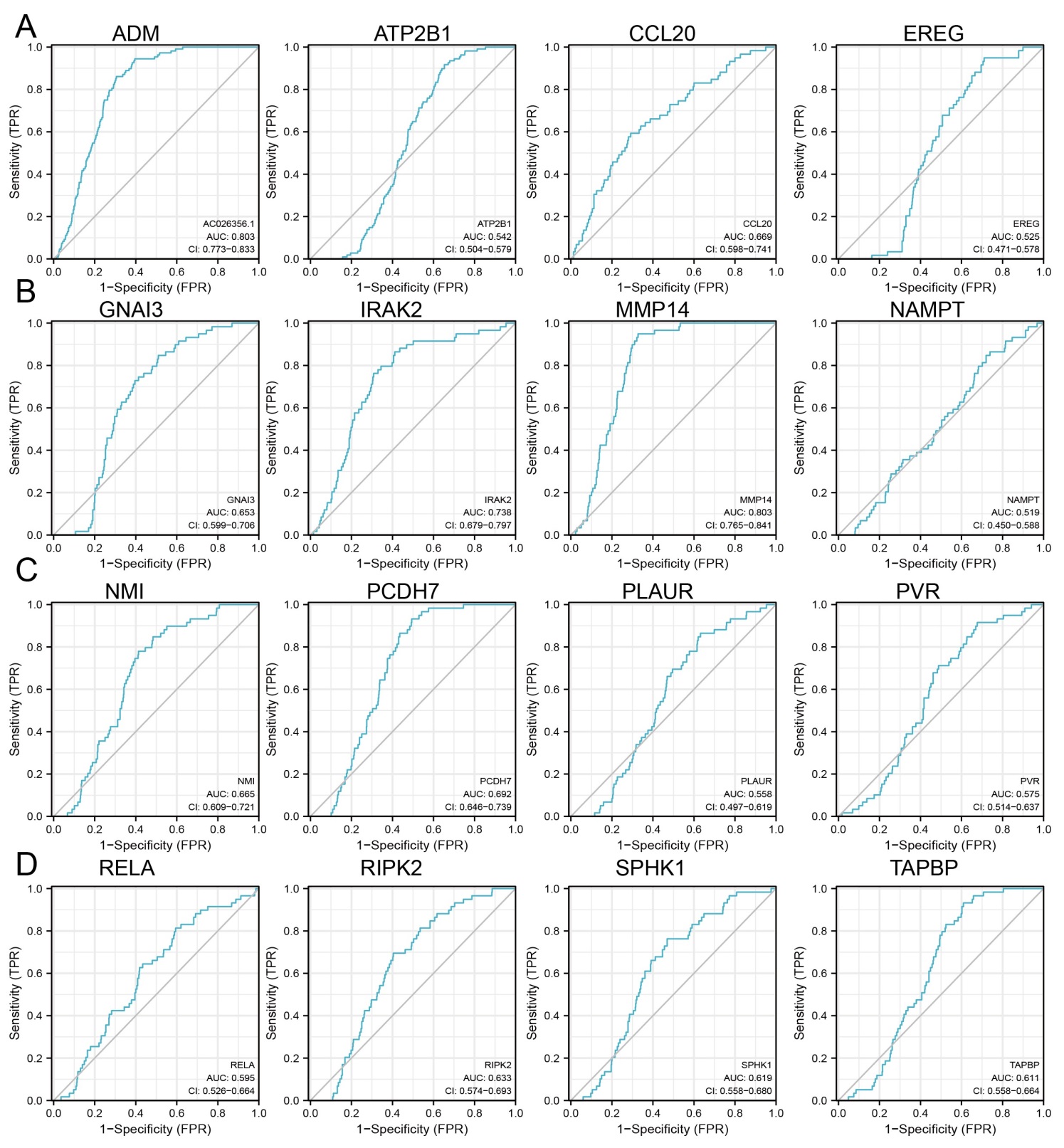
**

**Supplementary FIGURE 3 Analysis of the diagnosis by ROC curve for IRRGs in LUAD.**

(A-D) ROC curve analyses and AUC values for IRRGs in lung cancer.

**
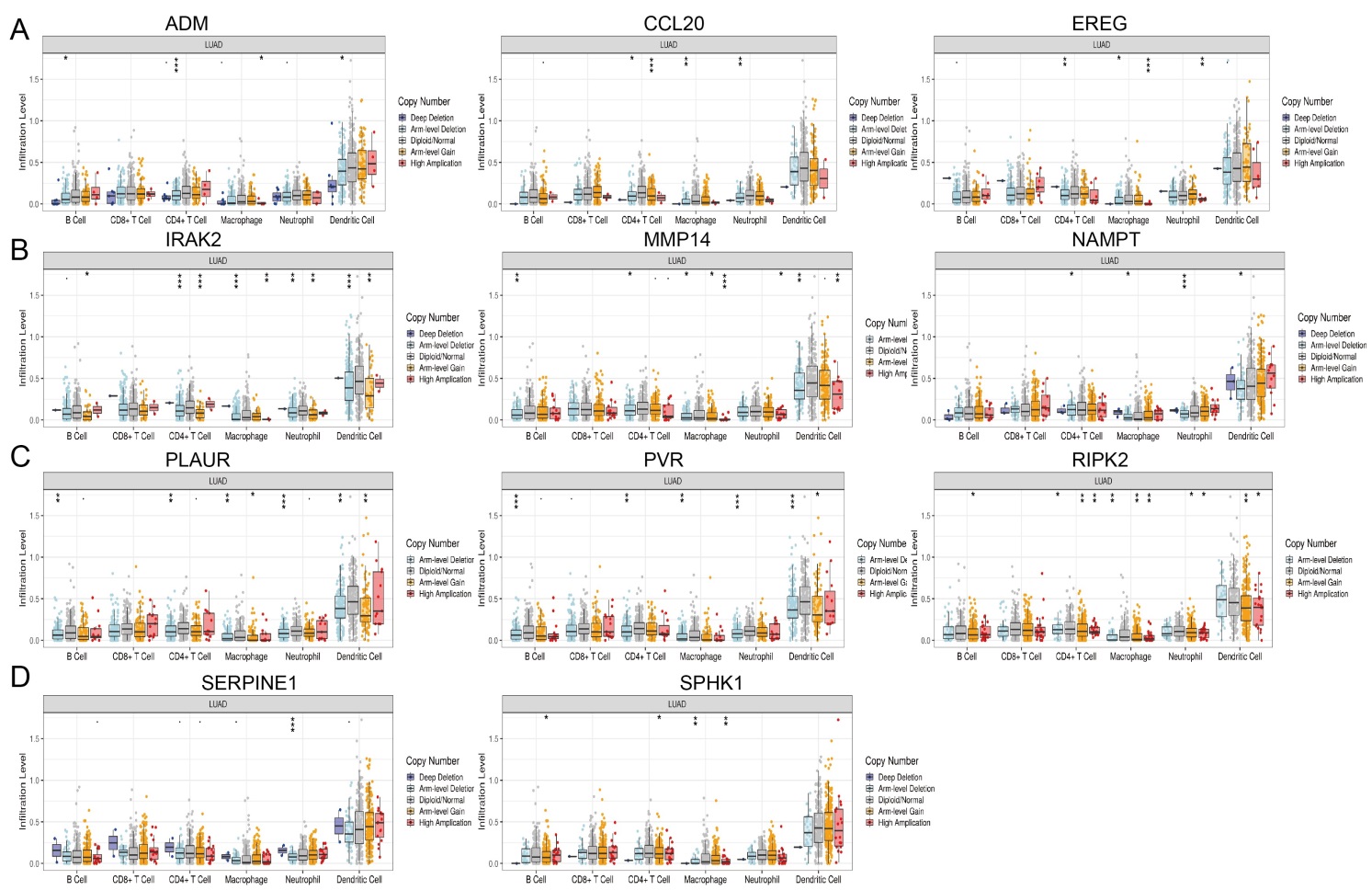
**

**Supplementary FIGURE 4 Analysis of the correlation between the tumor infiltrating levels in LUAD and somatic copy number alterations of IRRGs.**

(A-D) Correlation between tumor infiltrating levels and somatic copy number alterations of IRRGs in LUAD.

**
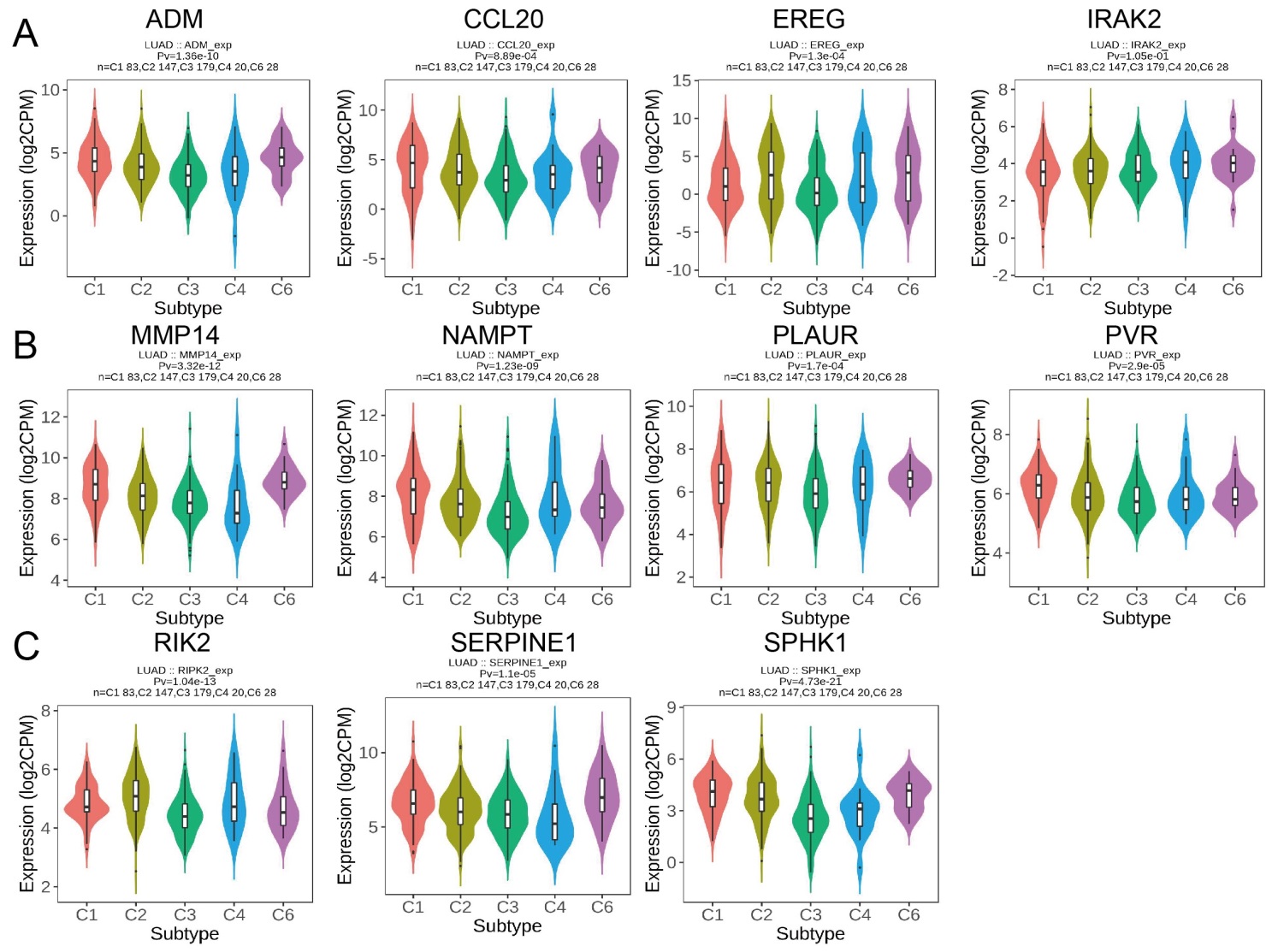
**

**Supplementary FIGURE 5 Analysis the expression of IRRGs** **immune subtype of LUAD.**

(A-C) The expression of IRRGs immune subtype of LUAD examined by TISIDB database.

**Supplementary Table 1 The prognosis of IRRGs examined by GEO datasets.**

| Gene name | Dataset | Cancer type | Prognosis | p value | ln(HR) | HR [95% CI-low CI-upp] | |
| --- | --- | --- | --- | --- | --- | --- | --- |
| TPBG | GSE31210 | Lung cancer | OS | 0.0001417 | 1.30766 | 3.70 [1.89 - 7.25] | |
| TPBG | GSE31210 | Lung cancer | RFS | 0.0006077 | 0.848249 | 2.34 [1.44 - 3.79] | |
| TPBG | GSE11117 | Lung cancer | OS | 0.0007257 | 1.13109 | 3.10 [1.61 - 5.97] | |
| TPBG | MICHIGAN | Lung cancer | OS | 0.0015489 | 1.46252 | 4.32 [1.75 - 10.68] | |
| TPBG | GSE8894 | Lung cancer | RFS | 0.0194482 | 0.279521 | 1.32 [1.05 - 1.67] | |
| TPBG | GSE13213 | Lung cancer | OS | 0.073791 | 0.294401 | 1.34 [0.97 - 1.85] | |
| ADM | GSE31210 | Lung cancer | RFS | 3.48E-06 | 0.809055 | 2.25 [1.60 - 3.16] | |
| ADM | GSE31210 | Lung cancer | OS | 4.83E-05 | 0.940433 | 2.56 [1.63 - 4.03] | |
| ADM | MICHIGAN | Lung cancer | OS | 0.000348 | 1.11232 | 3.04 [1.65 - 5.59] | |
| ADM | GSE14814 | Lung cancer | DSS | 0.0062156 | -0.349438 | 0.71 [0.55 - 0.91] | |
| ADM | GSE14814 | Lung cancer | OS | 0.0342312 | -0.243385 | 0.78 [0.63 - 0.98] | |
| ADM | GSE31210 | Lung cancer | RFS | 3.48E-06 | 0.809055 | 2.25 [1.60 - 3.16] | |
| ADM | GSE31210 | Lung cancer | OS | 4.83E-05 | 0.940433 | 2.56 [1.63 - 4.03] | |
| ADM | MICHIGAN | Lung cancer | OS | 0.000348 | 1.11232 | 3.04 [1.65 - 5.59] | |
| ADM | GSE14814 | Lung cancer | DSS | 0.0062156 | -0.349438 | 0.71 [0.55 - 0.91] | |
| ADM | GSE14814 | Lung cancer | OS | 0.0342312 | -0.243385 | 0.78 [0.63 - 0.98] | |
| ATP2B1 | GSE31210 | Lung cancer | RFS | 0.0065458 | -1.10015 | 0.33 [0.15 - 0.74] | |
| ATP2B1 | GSE31210 | Lung cancer | OS | 0.0093384 | -1.38822 | 0.25 [0.09 - 0.71] | |
| ATP2B1 | jacob | Lung cancer | OS | 0.0311232 | 0.63808 | 1.89 [1.06 - 3.38] | |
| ATP2B1 | jacob | Lung cancer | OS | 0.0313126 | 0.606026 | 1.83 [1.06 - 3.18] | |
| ATP2B1 | GSE31210 | Lung cancer | RFS | 0.0065458 | -1.10015 | 0.33 [0.15 - 0.74] | |
| ATP2B1 | GSE31210 | Lung cancer | OS | 0.0093384 | -1.38822 | 0.25 [0.09 - 0.71] | |
| ATP2B1 | jacob | Lung cancer | OS | 0.0311232 | 0.63808 | 1.89 [1.06 - 3.38] | |
| ATP2B1 | jacob | Lung cancer | OS | 0.0313126 | 0.606026 | 1.83 [1.06 - 3.18] | |
| CCL20 | GSE31210 | Lung cancer | RFS | 1.23E-06 | 0.445983 | 1.56 [1.30 - 1.87] | |
| CCL20 | GSE31210 | Lung cancer | OS | 0.000292 | 0.43654 | 1.55 [1.22 - 1.96] | |
| CCL20 | GSE3141 | Lung cancer | OS | 0.0411037 | 0.130249 | 1.14 [1.01 - 1.29] | |
| EREG | jacob | Lung cancer | OS | 0.0039436 | 0.315032 | 1.37 [1.11 - 1.70] | |
| EREG | GSE3141 | Lung cancer | OS | 0.0061739 | 0.224551 | 1.25 [1.07 - 1.47] | |
| EREG | GSE14814 | Lung cancer | OS | 0.0192436 | 0.343199 | 1.41 [1.06 - 1.88] | |
| EREG | GSE14814 | Lung cancer | DSS | 0.0203951 | 0.374474 | 1.45 [1.06 - 2.00] | |
| GNAI3 | GSE31210 | Lung cancer | RFS | 3.05E-12 | 4.29324 | 73.20 [21.91 - 244.60] | |
| GNAI3 | GSE31210 | Lung cancer | OS | 1.20E-09 | 5.16057 | 174.26 [33.01 - 919.92] | |
| GNAI3 | GSE31210 | Lung cancer | RFS | 4.67E-07 | 2.30881 | 10.06 [4.10 - 24.70] | |
| GNAI3 | GSE13213 | Lung cancer | OS | 1.50E-05 | 1.01947 | 2.77 [1.75 - 4.40] | |
| GNAI3 | GSE31210 | Lung cancer | OS | 0.0001166 | 2.3507 | 10.49 [3.17 - 34.69] | |
| GNAI3 | GSE8894 | Lung cancer | RFS | 0.0003593 | 0.768457 | 2.16 [1.41 - 3.29] | |
| GNAI3 | GSE31210 | Lung cancer | RFS | 0.0024224 | 1.45996 | 4.31 [1.68 - 11.06] | |
| GNAI3 | GSE8894 | Lung cancer | RFS | 0.0042543 | 0.439498 | 1.55 [1.15 - 2.10] | |
| GNAI3 | jacob | Lung cancer | OS | 0.0042933 | 1.66195 | 5.27 [1.68 - 16.49] | |
| GNAI3 | GSE8894 | Lung cancer | RFS | 0.0088284 | 0.638662 | 1.89 [1.17 - 3.05] | |
| GNAI3 | jacob | Lung cancer | OS | 0.0229005 | 0.836368 | 2.31 [1.12 - 4.74] | |
| GNAI3 | jacob | Lung cancer | OS | 0.0250394 | 1.10199 | 3.01 [1.15 - 7.89] | |
| GNAI3 | GSE3141 | Lung cancer | OS | 0.0328045 | 0.646311 | 1.91 [1.05 - 3.45] | |
| IRAK2 | GSE3141 | Lung cancer | OS | 0.0108724 | 0.613776 | 1.85 [1.15 - 2.96] | |
| IRAK2 | GSE31210 | Lung cancer | OS | 0.0213816 | 0.533291 | 1.70 [1.08 - 2.68] | |
| MMP14 | GSE31210 | Lung cancer | RFS | 2.24E-06 | 0.746729 | 2.11 [1.55 - 2.88] | |
| MMP14 | GSE31210 | Lung cancer | OS | 0.0028473 | 0.61719 | 1.85 [1.24 - 2.78] | |
| MMP14 | GSE3141 | Lung cancer | OS | 0.0041097 | 0.589537 | 1.80 [1.21 - 2.70] | |
| MMP14 | GSE8894 | Lung cancer | RFS | 0.0069945 | 0.441381 | 1.55 [1.13 - 2.14] | |
| MMP14 | GSE8894 | Lung cancer | RFS | 0.0093901 | 0.459743 | 1.58 [1.12 - 2.24] | |
| MMP14 | GSE3141 | Lung cancer | OS | 0.0154842 | 0.371215 | 1.45 [1.07 - 1.96] | |
| MMP14 | GSE8894 | Lung cancer | RFS | 0.0231333 | 0.311898 | 1.37 [1.04 - 1.79] | |
| MMP14 | GSE31210 | Lung cancer | RFS | 0.0484009 | 0.753288 | 2.12 [1.01 - 4.49] | |
| NAMPT | GSE3141 | Lung cancer | OS | 0.0153842 | 0.493629 | 1.64 [1.10 - 2.44] | |
| NAMPT | GSE31210 | Lung cancer | RFS | 0.0220213 | 0.458229 | 1.58 [1.07 - 2.34] | |
| NAMPT | MICHIGAN | Lung cancer | OS | 0.0246363 | 0.747329 | 2.11 [1.10 - 4.05] | |
| NAMPT | GSE3141 | Lung cancer | OS | 0.0317538 | 0.190183 | 1.21 [1.02 - 1.44] | |
| NAMPT | HARVARD | Lung cancer | OS | 0.0387324 | 0.323455 | 1.38 [1.02 - 1.88] | |
| NAMPT | GSE3141 | Lung cancer | OS | 0.0153842 | 0.493629 | 1.64 [1.10 - 2.44] | |
| NAMPT | GSE31210 | Lung cancer | RFS | 0.0220213 | 0.458229 | 1.58 [1.07 - 2.34] | |
| NAMPT | MICHIGAN | Lung cancer | OS | 0.0246363 | 0.747329 | 2.11 [1.10 - 4.05] | |
| NAMPT | GSE3141 | Lung cancer | OS | 0.0317538 | 0.190183 | 1.21 [1.02 - 1.44] | |
| NAMPT | HARVARD | Lung cancer | OS | 0.0387324 | 0.323455 | 1.38 [1.02 - 1.88] | |
| NMI | GSE31210 | Lung cancer | RFS | 1.14E-06 | 1.86325 | 6.44 [3.04 - 13.65] | |
| NMI | GSE31210 | Lung cancer | OS | 0.0006465 | 1.74646 | 5.73 [2.10 - 15.64] | |
| NMI | jacob | Lung cancer | OS | 0.0089535 | 1.14387 | 3.14 [1.33 - 7.40] | |
| PCDH7 | jacob | Lung cancer | OS |  | 0.00242238 | 0.374286 | 1.45 [1.14 - 1.85] |
| PCDH7 | jacob | Lung cancer | OS |  | 0.0036839 | 0.312906 | 1.37 [1.11 - 1.69] |
| PCDH7 | jacob | Lung cancer | OS |  | 0.00418302 | 0.413819 | 1.51 [1.14 - 2.01] |
| PCDH7 | jacob | Lung cancer | OS |  | 0.0103848 | 0.586361 | 1.80 [1.15 - 2.81] |
| PCDH7 | HARVARD | Lung cancer | OS |  | 0.0121879 | 0.411406 | 1.51 [1.09 - 2.08] |
| PCDH7 | jacob | Lung cancer | OS |  | 0.013499 | 0.165859 | 1.18 [1.03 - 1.35] |
| PCDH7 | jacob | Lung cancer | OS |  | 0.016483 | 0.246213 | 1.28 [1.05 - 1.56] |
| PCDH7 | jacob | Lung cancer | OS |  | 0.0231252 | 0.146416 | 1.16 [1.02 - 1.31] |
| PCDH7 | jacob | Lung cancer | OS |  | 0.0234982 | 0.244313 | 1.28 [1.03 - 1.58] |
| PCDH7 | jacob | Lung cancer | OS |  | 0.0271488 | 0.399177 | 1.49 [1.05 - 2.12] |
| PCDH7 | jacob | Lung cancer | OS |  | 0.037244 | -0.23836 | 0.79 [0.63 - 0.99] |
| PCDH7 | jacob | Lung cancer | OS |  | 0.0374481 | 0.351863 | 1.42 [1.02 - 1.98] |
| PCDH7 | jacob | Lung cancer | OS |  | 0.0449027 | 0.174103 | 1.19 [1.00 - 1.41] |
| PLAUR | GSE31210 | Lung cancer | RFS | 1.72E-06 | 0.833065 | 2.30 [1.64 - 3.24] | |
| PLAUR | GSE31210 | Lung cancer | RFS | 1.01E-05 | 0.811369 | 2.25 [1.57 - 3.23] | |
| PLAUR | GSE31210 | Lung cancer | RFS | 2.92E-05 | 0.750125 | 2.12 [1.49 - 3.01] | |
| PLAUR | GSE31210 | Lung cancer | OS | 3.59E-05 | 0.913904 | 2.49 [1.62 - 3.85] | |
| PLAUR | GSE31210 | Lung cancer | OS | 5.73E-05 | 0.926953 | 2.53 [1.61 - 3.97] | |
| PLAUR | GSE31210 | Lung cancer | OS | 0.0001054 | 0.873312 | 2.39 [1.54 - 3.72] | |
| PLAUR | GSE3141 | Lung cancer | OS | 0.0005891 | 0.564734 | 1.76 [1.27 - 2.43] | |
| PLAUR | GSE8894 | Lung cancer | RFS | 0.0009977 | 0.35654 | 1.43 [1.16 - 1.77] | |
| PLAUR | GSE8894 | Lung cancer | RFS | 0.0018653 | 0.399485 | 1.49 [1.16 - 1.92] | |
| PLAUR | GSE8894 | Lung cancer | RFS | 0.0035398 | 0.285125 | 1.33 [1.10 - 1.61] | |
| PLAUR | jacob-UM | Lung cancer | OS | 0.0043135 | -0.647541 | 0.52 [0.34 - 0.82] | |
| PLAUR | jacob | Lung cancer | OS | 0.0324191 | 0.8054 | 2.24 [1.07 - 4.68] | |
| PLAUR | HARVARD | Lung cancer | OS | 0.0334299 | -1.46381 | 0.23 [0.06 - 0.89] | |
| PLAUR | GSE14814 | Lung cancer | DSS | 0.0357534 | 0.900196 | 2.46 [1.06 - 5.70] | |
| PLAUR | GSE3141 | Lung cancer | OS | 0.039402 | 0.440127 | 1.55 [1.02 - 2.36] | |
| PLAUR | GSE3141 | Lung cancer | OS | 0.0484045 | 0.236955 | 1.27 [1.00 - 1.60] | |
| PVR | jacob | Lung cancer | OS | 0.0012537 | 0.736764 | 2.09 [1.34 - 3.27] | |
| PVR | jacob | Lung cancer | OS | 0.0030283 | 1.20745 | 3.34 [1.51 - 7.43] | |
| PVR | jacob | Lung cancer | OS | 0.0054314 | 0.52807 | 1.70 [1.17 - 2.46] | |
| PVR | jacob | Lung cancer | OS | 0.006755 | 0.895162 | 2.45 [1.28 - 4.68] | |
| PVR | jacob | Lung cancer | OS | 0.0076716 | 0.662853 | 1.94 [1.19 - 3.16] | |
| PVR | HARVARD | Lung cancer | OS | 0.0088724 | 0.830553 | 2.29 [1.23 - 4.27] | |
| PVR | jacob | Lung cancer | OS | 0.012838 | 1.04077 | 2.83 [1.25 - 6.43] | |
| PVR | jacob | Lung cancer | OS | 0.0130375 | 1.14749 | 3.15 [1.27 - 7.79] | |
| PVR | jacob | Lung cancer | OS | 0.0131302 | -0.752257 | 0.47 [0.26 - 0.85] | |
| PVR | jacob | Lung cancer | OS | 0.0148388 | 0.586049 | 1.80 [1.12 - 2.88] | |
| PVR | jacob | Lung cancer | OS | 0.0186442 | 0.643831 | 1.90 [1.11 - 3.26] | |
| PVR | jacob | Lung cancer | OS | 0.0249147 | 1.43616 | 4.20 [1.20 - 14.75] | |
| PVR | jacob | Lung cancer | OS | 0.0342158 | 0.637468 | 1.89 [1.05 - 3.41] | |
| PVR | jacob | Lung cancer | OS | 0.040333 | 0.412159 | 1.51 [1.02 - 2.24] | |
| PVR | jacob | Lung cancer | OS | 0.0422555 | 0.687607 | 1.99 [1.02 - 3.86] | |
| RELA | MICHIGAN | Lung cancer | OS | 0.0098308 | 2.64506 | 14.08 [1.89 - 104.91] | |
| RIPK2 | GSE31210 | Lung cancer | RFS | 4.93E-08 | 1.42701 | 4.17 [2.49 - 6.96] | |
| RIPK2 | GSE31210 | Lung cancer | OS | 2.68E-05 | 1.44235 | 4.23 [2.16 - 8.29] | |
| RIPK2 | GSE31210 | Lung cancer | OS | 0.0102787 | 1.01379 | 2.76 [1.27 - 5.98] | |
| RIPK2 | GSE13213 | Lung cancer | OS | 0.0170953 | 0.483303 | 1.62 [1.09 - 2.41] | |
| RIPK2 | jacob-UM | Lung cancer | OS | 0.0457582 | 0.597276 | 1.82 [1.01 - 3.27] | |
| RIPK2 | GSE14814 | Lung cancer | OS | 0.0479337 | 1.87103 | 6.49 [1.02 - 41.47] | |
| SERPINE1 | GSE4716 | Lung cancer | OS | 0.0162522 | 0.740672 | 2.10 [1.15 - 3.84] | |
| SERPINE1 | MICHIGAN | Lung cancer | OS | 0.0173498 | 0.778354 | 2.18 [1.15 - 4.14] | |
| SERPINE1 | GSE3141 | Lung cancer | OS | 0.0181219 | 0.406141 | 1.50 [1.07 - 2.10] | |
| SERPINE1 | GSE31210 | Lung cancer | RFS | 0.0212839 | -0.384705 | 0.68 [0.49 - 0.94] | |
| SERPINE1 | jacob | Lung cancer | OS | 0.027686 | 0.419549 | 1.52 [1.05 - 2.21] | |
| SERPINE1 | GSE3141 | Lung cancer | OS | 0.0300864 | 0.288755 | 1.33 [1.03 - 1.73] | |
| SERPINE1 | GSE8894 | Lung cancer | RFS | 0.0321311 | 0.153413 | 1.17 [1.01 - 1.34] | |
| SERPINE1 | jacob | Lung cancer | OS | 0.0482273 | 0.234875 | 1.26 [1.00 - 1.60] | |
| SPHK1 | GSE31210 | Lung cancer | RFS | 1.46E-05 | 0.826979 | 2.29 [1.57 - 3.32] | |
| SPHK1 | GSE31210 | Lung cancer | OS | 0.003255 | 0.772523 | 2.17 [1.29 - 3.62] | |
| TAPBP | GSE31210 | Lung cancer | RFS | 0.0064462 | 0.473469 | 1.61 [1.14 - 2.26] | |
| TAPBP | GSE31210 | Lung cancer | OS | 0.0353141 | 1.38984 | 4.01 [1.10 - 14.65] | |
| TAPBP | jacob | Lung cancer | OS | 0.0363128 | -0.815899 | 0.44 [0.21 - 0.95] | |
